# SMYD2 promotes cervical cancer growth by stimulating cell proliferation

**DOI:** 10.1186/s13578-019-0340-9

**Published:** 2019-09-18

**Authors:** Jun-Jie Sun, Hong-Lin Li, Hui Ma, Yang Shi, Li-Rong Yin, Su-Jie Guo

**Affiliations:** Department of Gynecology, The Secondary Hospital of Tianjin Medical University, No. 23 Pingjiang Road, Hexi District, Tianjin, 300211 China

**Keywords:** Cervical cancer, SMYD2, Prognosis, Proliferation, Therapeutic target

## Abstract

**Background:**

Cervical cancer is the most common gynecological malignancy with low terminal cure rate, and therefore new therapeutic targets are urgently needed to combat this disease. SMYD2, as an oncogene, is abnormal highly expressed in multiple types of tumors and further affects the occurrence and development, but the potential correlations between SMYD2 expression and cervical cancer progression is still unclear.

**Methods:**

We first used the bioinformatics website to screen the data of cervical cancer in (The Cancer Genome Atlas) TCGA and survival analysis was used to find the different survival rates in the SMYD2 high expression group and low expression group. Through immunohistochemistry, the association between SMYD2 expression and clinical-pathological features of cervical cancer patients was further evaluated. Quantitative PCR and Immunoblot were applied to investigate the relative mRNA and protein expression levels, respectively. In vivo and in vitro experiments were performed to explore the function of SMYD2 in cancer progression.

**Results:**

We first found a high expression of SMYD2 in cervical cancer, and survival analysis found that the poorer survival rate in the SMYD2 high expression group than that in the low expression group. Herein, our study demonstrated that the expression of SMYD2 in patients with cervical cancer was associated with FIGO stage, tumor size and further correlated with poor prognosis. Our data further showed that the inhibition of SMYD2 expression in cervical cancer cell line Caski and Siha could dramatically block the proliferation of cervical cancer cells. Additionally, SMYD2-shRNA lentivirus infected remarkably inhibited tumorigenesis in mice compared with the scramble group.

**Conclusions:**

Taken together, this study provides strong evidence of the involvement of SMYD2 in cervical cancer growth and indicates that it could have high potential as a therapeutic target of cervical cancer.

## Background

Cervical cancer is the most common gynecological malignancy [1]. In recent years, the incidence of the disease has a younger trend [2]. With the discovery of multiple cervical cancer bio-markers, cervical cancer can be detected and treated early, and the incidence and mortality of cervical cancer have been significantly reduced [3, 4]. At present, the treatment of cervical cancer mainly focuses on surgery and radiotherapy, but the survival rates of patients with advanced stage is very low, and the 5-year survival rates of invasive cervical cancer is only 67% [5, 6]. Additionally, early cervical cancer has no obvious symptoms, which therefore increases the difficulty of diagnosis [7]. In order to combat this disease, not only improved early diagnosis is requirement, novel and promising therapeutic targets are also urgly needed [8].

SET and MYND domain-containing protein 2 (SMYD2) is a lysine methyltransferase, which could methylate H3K36 and participate in transcriptional regulation [9]. SMYD2 is reported to regulate the differentiation of muscle cells and closely related to the formation and development of cardiovascular, nervous, and reproductive systems [10–12]. As an oncogene, SMYD2 is highly expressed in a variety of human tumors such as bladder cancer and gastric cancer, and the high expression of SMYD2 is closely related to the poor prognosis of patients with these diseases [13, 14]. In addition, evidences indicate that SMYD2 could improve the activity of the poly (ADP-ribose) of PARP1, an oncogenic protein, and block the function of p53 and PTEN in cancer cells [15–17]. Previous data also demonstrated that SMYD2-mediated ALK methylation dramatically promoted lung cancer development, and SMYD2 was also involved the growth of MLL-AF9 induced leukemogenesis [18, 19]. Although numerous studies had confirmed the critical role of SMYD2 in the development and progression of tumors, there were few reports on whether SMYD2 affected cervical cancer progression.

In this study, both the TCGA data and our finding confirmed that SMYD2 was positively correlated with poor prognosis of the patients with cervical cancer. SMYD2, in addition, was found to be associated with FIGO stage and tumor size of cervical cancer. SMYD2 ablation in cervical cancer cells Caski and Siha obviously inhibited cell proliferation and suppressed tumor formation in vivo. Therefore, SMYD2 could represent as a potential and novel therapeutic target for the treatment of cervical cancer.

## Materials and methods

### Antibodies, primers and plasmids

Anti-SMYD2 (for Immunohistochemical, 1:100 dilution, ab234862, abcam; for Immunoblot, 1:5000 dilution, ab108217, abcam), anti-β-actin (1:2000 dilution, mAb #3700, CST), anti-Ki67 (1:1000 dilution, 27309-1-AP, Proteintech), anti-proliferating cell nuclear antigen (PCNA) (1:500 dilution, SAB2108448, Sigma-Aldrich).

The qRT-PCR primer sequences of SMYD2 as follows: forward, 5′-ACAGGAAATCAAGCCGGGAG-3′ and reverse, 5′-GGTACACTCCTGGCACTCAC-3′; The qRT-PCR primer sequences of GAPDH are as follows: 5′-CGACCACTTTGTCAAGCTCA-3′ and 5′-GGTTGAGCACAGGGTACTTTATT-3′. Ready-to-package AAV shRNA clone of SMYD2 was bought from the Addgene plc.

### Human tissue samples

85 patients clinically and pathologically diagnosed with papillary cervical cancer in the secondary hospital of Tianjin medical university were collected in this study. Tumor tissues were obtained after the surgical treatment. The clinical characters, such as ages, genders, pTMN stage, tumor size, and lymph metastasis of patient were recorded.

To further explore the link between SMYD2 and cervical cancer, immunohistochemical assays were then performed. SMYD2 is mainly located in the cytoplasm of cervical cancer cells, which is brownish yellow and granular. Samples was classified into four groups according to the staining intensity (0 = negative; 1 = low; 2 = medium; 3 = high). Meanwhile, the percentage of stained tumor cells was below: 0 = 0% stained cells; 1 = 1–25% stained cells; 2 = 26–50% stained cells; 3 = 51–100% stained cells. And the score of staining intensity × the score of stained cells percentage < 2 was considered as negative staining, 2–3 was considered as low staining and > 4 was considered as high staining. The results were judged by double-blind method.

### Cell culture and transfection

The Caski and Siha human cervical cancer cells were purchased from ATCC (Chicago, USA). Caski cells were cultured in RPMI-1640 culture medium, and Siha cells were maintained in EMEM culture medium, supplemented with 10% of fetal bovine serum. Both of them were incubated at 37 °C in a 5% CO _2_ incubator.

The SMYD2 shRNA plasmids were transfected into cervical cancer cells by Invitrogen Lipofectamine^®^ 2000 (Thermo Fisher Scientific, Inc.). The specific shRNA with the sequence of AAAGAAGGATTGTCCAAATGTGG (Cat# SH843615, vigene biosciences, Rockville, USA) to target SMYD2, and scrambled sequence was used as negative control. 100,000 cell per well in six-well plates according to the manufacturer’s protocol, 3 groups were set, including: sh-SMYD2 group, which transfected with shRNA targeting SMYD2; Negative Control group, which transfected with scrambled sequence; and Mock group was treated without transfection (data not shown). Silence-efficiency was measured by RT-PCR and Western Blot after 48 h transfection. Then the SMYD2 stable depletion cell lines was screened and used for the in vitro and in vivo assays.

### Quantitative-PCR assay

Total RNA was extracted from cervical cancer cells by Trizol reagent (Invitrogen). Subsequently total mRNA was reverse-transcribed by M-MLV reverse transcriptase (Promega). Quantitative PCR was conducted using SYBR mixture (Takara), and the relative expression level of SMYD2 was normalized to GAPDH.

### Immunoblot assay

Samples extracted from cervical cancer cells or tissues were separated by SDS-PAGE, sequentially transferred onto nitrocellulose membranes, followed by blocking with 5% fat-free milk. Membranes were then incubated with primary antibodies targeting SMYD2, Ki67, PCNA, and β-actin for 2 h. Then the membranes were incubated with HRP-conjugate secondary antibodies for 45 min. Signals were visualized by an ECL kit.

### Colony formation assay

Both Caski and Siha cells were resuspended and plated in 6-well plates with a density of 2000 cells per well and grown for 14 days. The cells were fixed with 4% paraformaldehyde for 20 min and stained with 0.2% crystal violet for 20 min. Photographs were then taken and the difference of colony numbers between shControl and shSMYD2 cervical cancer cells were explored.

### MTT assay

Caski and Siha cells were plated in 96-well plates with a density of 1000 cells per well and cultured for 48 h. Cells were then treated with MTT for 4 h and washed with PBS. Cells were then extracted by 150-μL DMSO and the OD value at a wavelength of 570 nm was measured.

### Tumor growth in vivo

The operation of mice was approved by our Institutional Animal Care Committee. Nude BalB/c mice (6–8 weeks, 18–22 g) were purchased from Beijing Vital River Laboratory Animal Technology Co., Ltd. (Beijing, China). To measure tumor volume in vivo, Caski cervical cancer cells were stably infected with control or SMYD2 shRNA lentivirus and injected subcutaneously into the right flank of female nude mice. Nearly 2 weeks later, tumors (150 mm^3^) were established, and the tumor volume was measured per week and calculated in (length × width^2^)/2. And the results of IHC was analyzed below: The sections of each group were observed within 5 visual fields, and the positive cells number was counted. Then we could obtain the mean number and SD.

### Statistics

Data were analyzed with SPSS 22.0 software. For the immunohistochemistry experiments, associations between SMYD2 expression and the clinicopathological features were evaluated using χ^2^ tests. Associations of survival and tumor progression and SMYD2 expression were estimated by Kaplan–Meier method and log-rank tests. Data are shown as the mean ± standard deviation (SD) in vitro and in vivo experiments. Student’s t-test was used for statistical comparisons. A value of P < 0.05 was setted to be statistically significant.

## Results

### Bioinformatic analysis of SMYD2 in cervical cancer patients

**Fig. 1:**
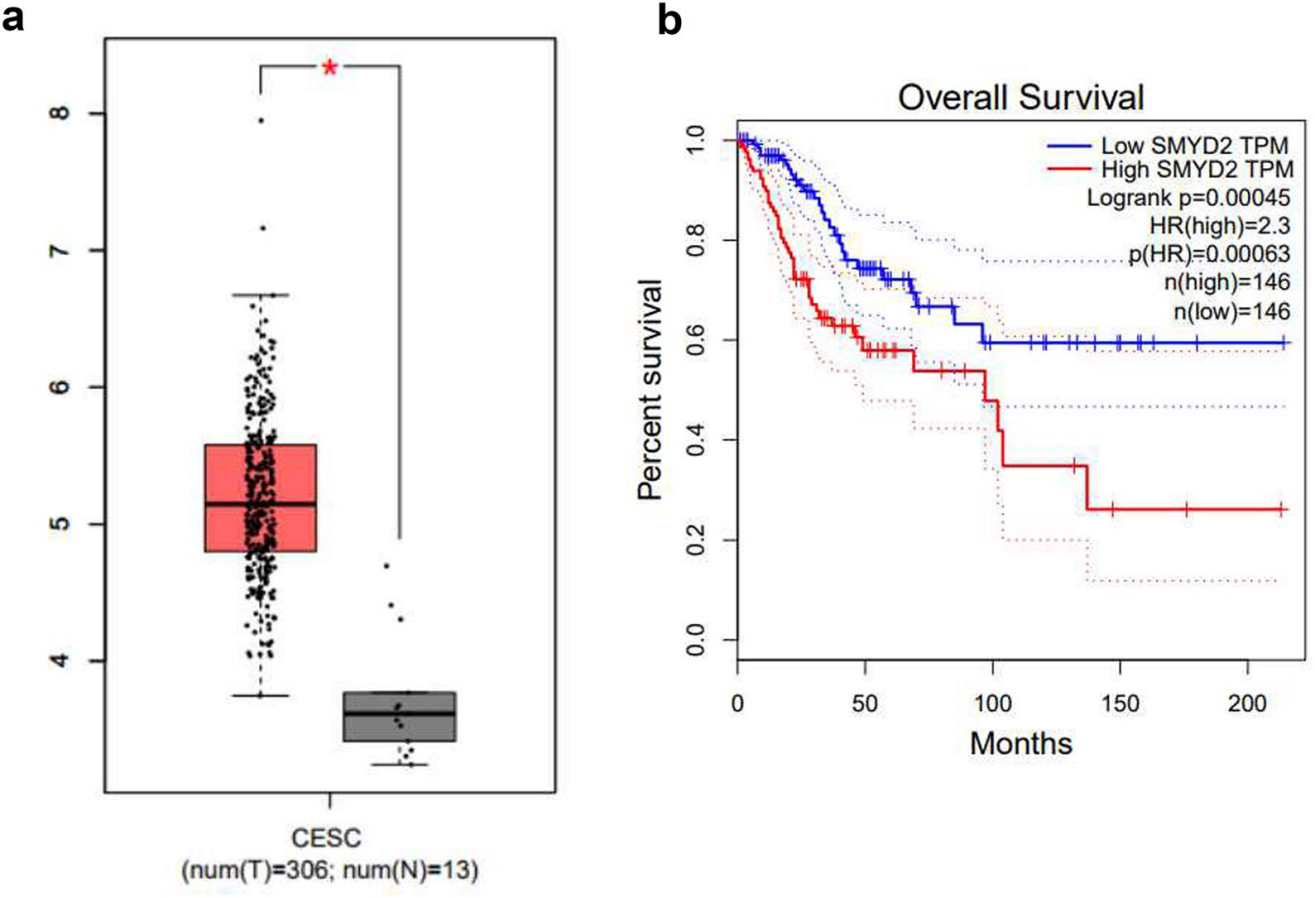
Bioinformatic analysis of SMYD2 in cases from TCGA. **a** The expression of SMYD2 between cancer (n = 306) and normal (n = 13) cases, cancer cases were filled in red, normal cases were in blue: SMYD2 expression was significantly increased in pancreatic tumor tissue compared with normal pancreatic tissue (*P *< 0.05). *P < 0.05. **b** The overall survival and disease free survival rate of 306 TCGA cases with clear clinical information. High and low groups were divided by median and filled in red and blue, respectively. Comparing with the lower expression cases, patients with higher level of SMYD2 shared a significantly worse overall survival (*P *= 0.00045 < 0.05)

Bioinformatic method were performed to analyze the expression of SMYD2 at mRNA levels in cervical cancer patients by using GEPIA, an online analytical database that analyze cases from TCGA, and we found that SMYD2 was abnormal highly expressed in the cervical cancer tissues compared with normal tissues (Fig. 1a, P < 0.05, http://gepia.cancer-pku.cn/detail.php?gene=SMYD2). A total of 306 cervical cancer cases of TCGA patients were analyzed, and the median SMYD2 mRNA expression was used as the cutoff point to divide all the patients into high and low expression groups according to the median. Comparing with the lower expression cases, patients with higher level of SMYD2 shared a significantly worse overall survival (Fig. 1b, P = 0.00045 < 0.05, http://gepia.cancer-pku.cn/detail.php?gene=SMYD2). These results give a clue of a potential oncogene role of SMYD2 in cervical cancer.

### SMYD2 is associated with the poor prognosis of cervical cancer patients

**Fig. 2:**
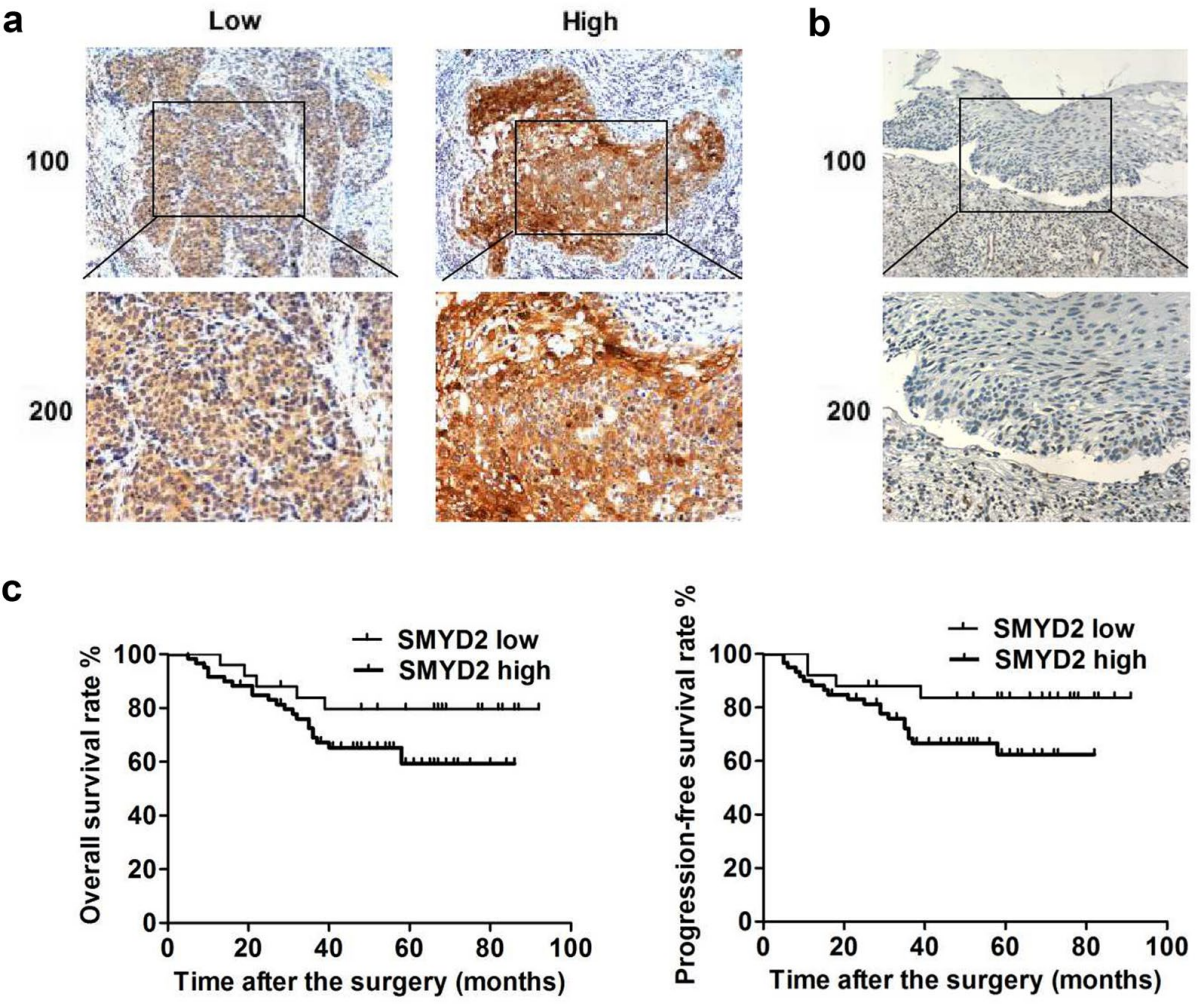
SMYD2 expression in cervical tumor tissues was associated with poor prognosis of patients. **a** Immunohistochemical assays were performed, and the representative images of SMYD2 expression in cervical cancer tissues were showed (×100 and ×200 magnification, respectively). **b** Immunostaining results of SMYD2 expression in the adjacent tissues (×100 and ×200 magnification, respectively). **c** The KM-Plot analysis of overall survival rate and disease-free survival rate between low and high-SMYD2 expression groups

To explore the potential link between SMYD2 and cervical cancer development, immunohistochemical assays were performed. The staining intensity of SMYD2 in clinical samples from 85 cervical cancer patients was then detected. Interestingly, results showed that SMYD2 was mainly localized in the cytoplasm and highly expressed in cervical tumor tissues (Fig. 2a). Based on the SMYD2 expression level, clinical samples are classified into SMYD2 low (70.6%) and high-expressed (29.4%) groups (Fig. 2a). Meanwhile, obvious low expression of SMYD2 was detected in adjacent tissues, providing evidences that SMYD2 could play a key role in the growth and development of cervical cancer (Fig. 2b).

According to the clinicopathological characteristic data on patients with cervical cancer, we analyzed the difference between low and high expression cervical groups. Interestingly, the expression of SMYD2 in the cervical tumors was remarkably associated with FIGO stage and tumor size (P < 0.05, respectively), indicating a potential relationship between SMYD2 and cervical cancer (Table 1). Whereas no obvious difference was found between high and low SMYD2 groups in other aspects, such as patient age and tumor grade (Table 1).

**Table 1:** Relationships of SMYD2 and clinicopathological characteristics in 85 patients with cervical cancer

Feature	All n = 85	SMYD2 expression		χ^2^	*P*
		High	Low		
		n = 60	n = 25		
Age (years)				0.400	0.708
< 40	45	30	15		
≥ 40	40	30	10		
FIGO stage				4.896	0.027*
IB1	42	25	17		
IB2-IIB	43	35	8		
Differentiation				2.539	0.111
Well/moderately	50	32	18		
Poorly	35	28	7		
Tumor size (cm)				5.773	0.016*
< 4	55	34	21		
≥ 4	30	26	4		

*Stand for *P* < 0.05

We also investigated the prognosis of cervical cancer patients in these two groups, and found that SMYD2 low-expression patients had higher overall survival rate and disease-free survival rate, compared with SMYD2 high-expression groups (Fig. 2c). Collectively, these results revealed that SMYD2 was associated with the poor prognosis of cervical cancer patients.

### Knockdown of SMYD2 blocked the proliferation of cervical cancer in vitro

**Fig. 3:**
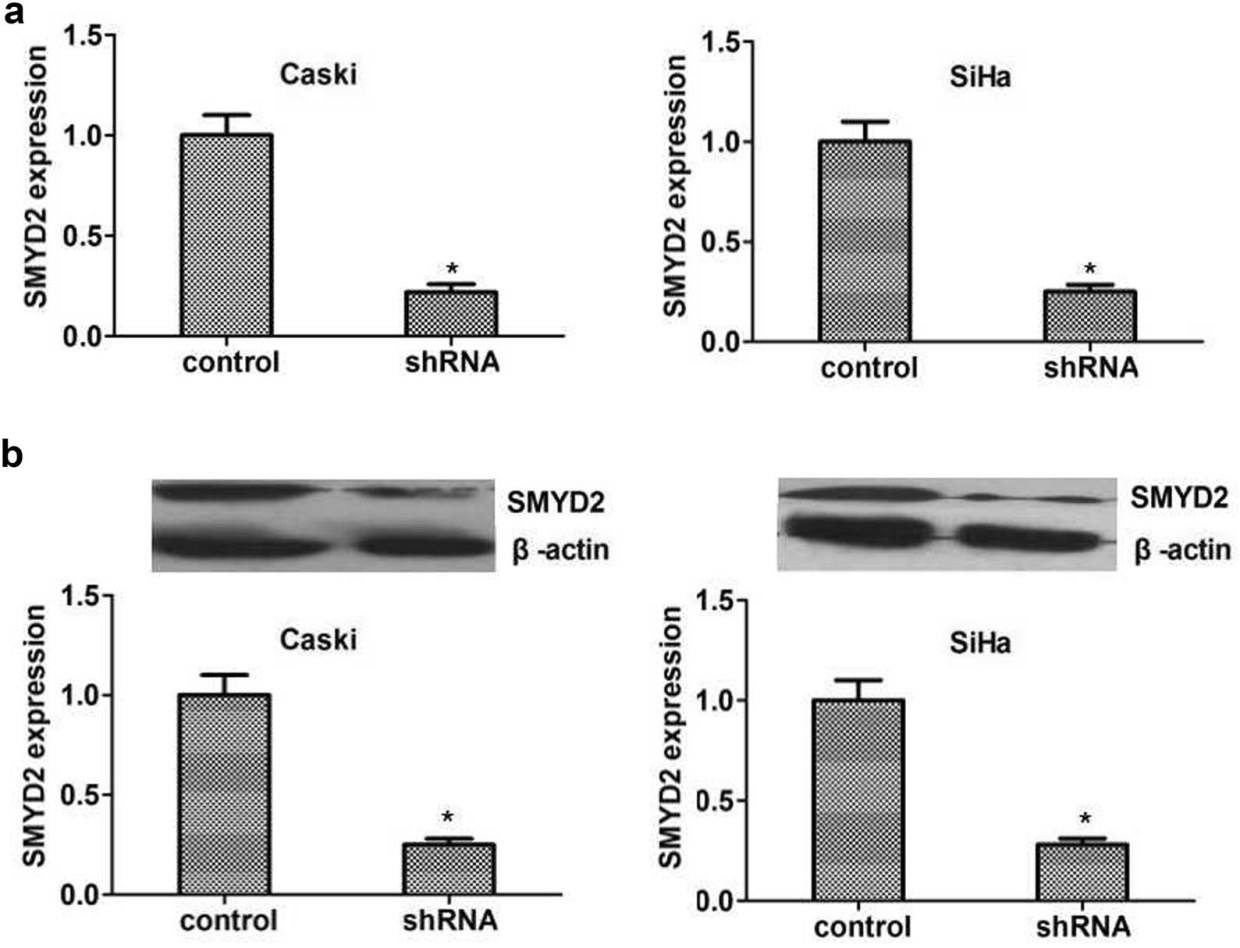
SMYD2 was down-regulated effectively in two types of human cervical cancer cells caused by its shRNA. **a** Results of qRT-PCR assay showed the expression level of SMYD2 was sufficiently knockdown in the 2 types of cervical cancer cells, respectively. **b** Results of Immunoblot revealed the efficiently silenced of SMYD2 expression in both Caski and Siha cells. Results are presented as mean ± SD, *P < 0.05

To further explore the mechanism of SMYD2 affecting cervical cancer, we used SMYD2-targeted shRNA to inhibit its expression in two types of cervival cancer cells including Siha and Caski cells. The silencing efficiency mediated by SMYD2 shRNA was detected simultaneously by both quantitative PCR and immuneblot assays. Results revealed that SMYD2 shRNA was enough to significantly decrease the expression of SMYD2 in both mRNA and protein level, respectively (Fig. 3a, b).

**Fig. 4:**
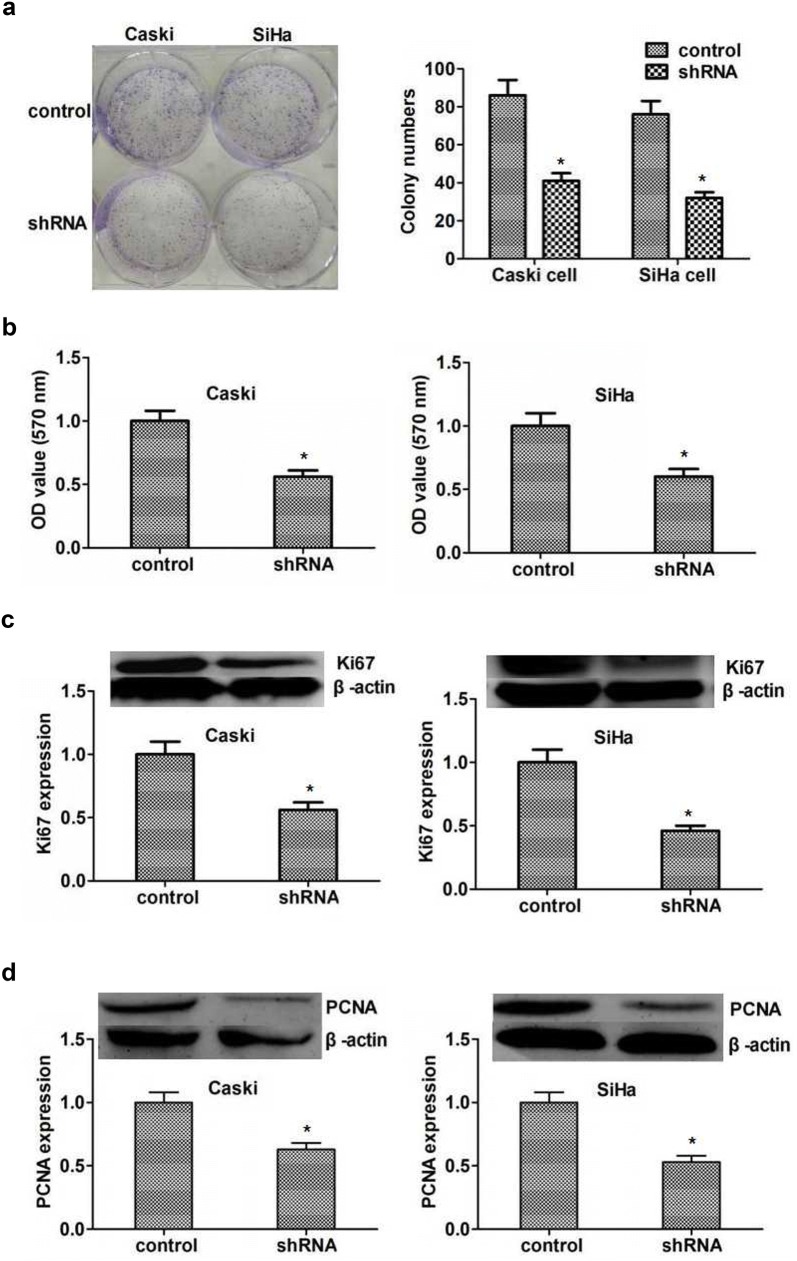
SMYD2 depletion obviously restrained the proliferation of cervical cancer cells. **a** Representive images showed the results of colony formation assays of Caski and Siha cells that were transfected with control or SMYD2 shRNA. Cultivation lasted 2 weeks. **b** MTT assays showed the difference between control and SMYD2 knockdown cervical cancer cells. **c** Immunoblot assay revealed the markedly decreased expression of SMYD2 in both Caski and Siha cells. **d** Immunoblot assay showed the PCNA expression was obviously down-regulated in 2 types of cervical cancer cells. Results are presented as mean ± SD, *P < 0.05

On this basis, we then examined the effect of SMYD2 on proliferation of cervical cancer cells performing colony formation and MTT assays. As we expected, the proliferation ability was dramatically restrained by SMYD2 depletion, proved by the obviously decreased cell numbers (Fig. 4a). Additionally, the results of MTT assays showed an obvious decreased absorbance value at 570 nm wavelength in both Siha and Caski cells (Fig. 4b).

In general, the inhibition of cell proliferation could lead to decreased expression of proliferating cell marker, Ki67 and PCNA. To further clarify the role of SMYD2 in proliferation of cervical cancer cells in vitro, we examined the expression level of Ki67 and PCNA, respectively. A remarkably downregulated of ki67 and PCNA was found in SMYD2 depleted cervical cancer cells. These results confirmed the important role of SMYD2 in the regulation of cervical cancer proliferation (Fig. 4c, d).

### SMYD2 ablation impaired proliferation of cervical cancer in vivo

Since previous results suggested the potential effects of SMYD2 on the proliferation of cervical cancer in vivo, we next further explore the role of SMYD2 in the growth and development of cervical cancer in mice.

**Fig. 5:**
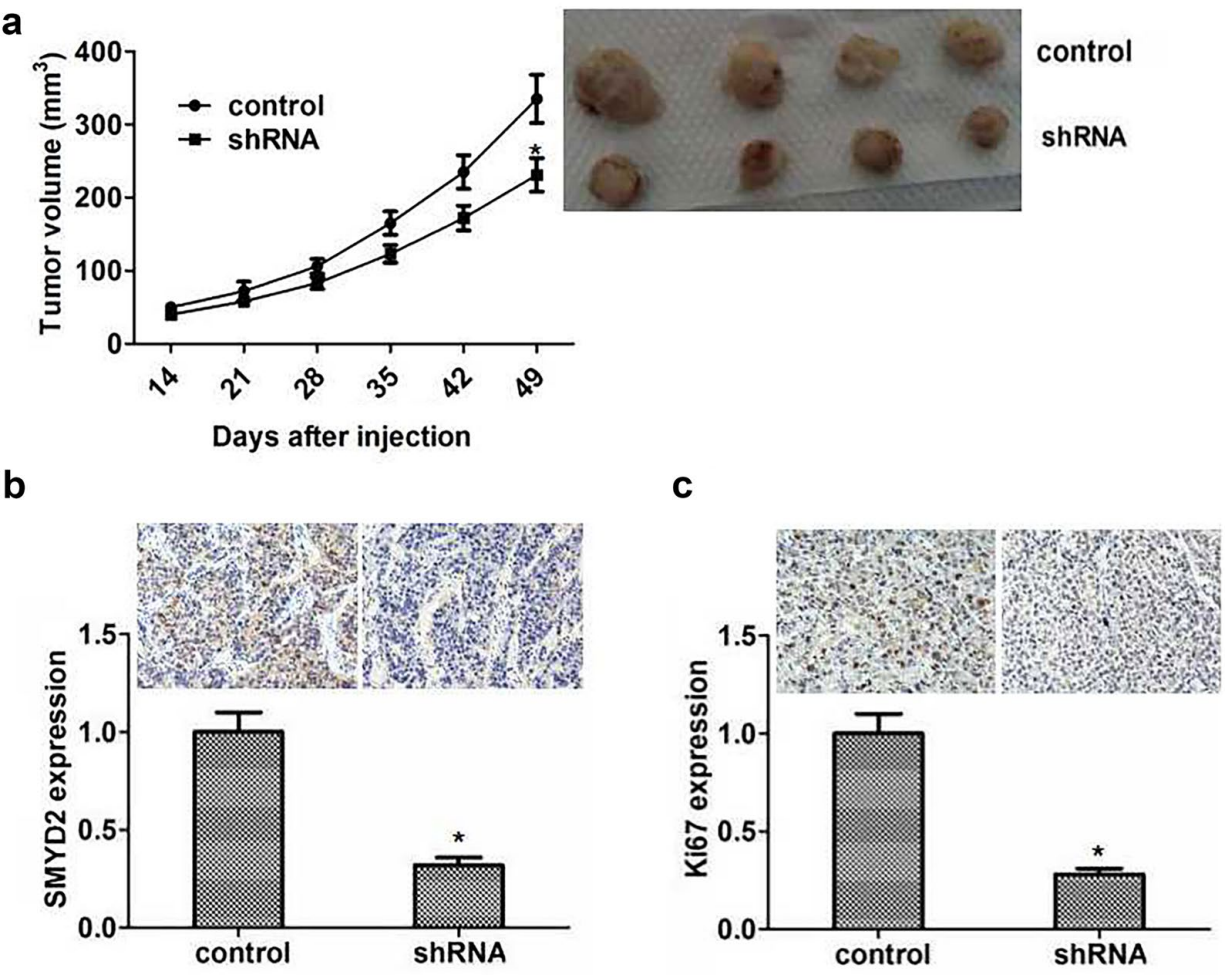
SMYD2 promotes cervical cancer growth in mice. **a** Caski cells were infected with SMYD2 or control shRNA lentivirus, subcutaneous implanted into nude mice. 2 weeks later, tumors were isolated and photographed per week. (n = 4 in each group). Tumor growth curves was calculated according to average volume of 4 tumors for each group. **b**, **c** The results of immunohistochemical indicated the difference of SMYD2 and Ki67 expression between control and SMYD2 ablation groups. Results are presented as mean ± SD, *P < 0.05

Caski cells were firstly infected with control and SMYD2 shRNA lentivirus and subsequently subcutaneous injected into nude mice. 2 weeks later, the volume of tumors was measured every week. Representative tumor photographs were taken and exhibited in Fig. 5a. Interestingly, the volume of tumors removed from SMYD2 depletion group mice was obviously smaller than control groups (Fig. 5a). We then detected the SMYD2 expression in tumor tissues of mice to confirm the silencing efficiency, and the results of immunohistochemistry assay confirmed a significant decrease expression of SMYD2 in SMYD2 knockdown tumor tissues of mice (Fig. 5b). Then we also found that the significant decrease expression of PCNA protein in the SMYD2 knockdown tumor tissues of mice (Fig. 5c). Therefore, all these data showed that SMYD2 was important in the regulation of cervical cancer proliferation.

## Discussion

As one of the most common gynecological tumors with a high degree of malignancy, screening and early diagnosis of cervical cancer have attracted more attention [20]. With the use of multiple effective cervical cancer biomarkers, such as SCC and CA125, the early diagnosis of cervical cancer has made significant progress [21, 22]. Up to now, for advanced invasive cervical cancer, the mortality rate is still high, and the existing therapeutic targets are still difficult to meet the treatment needs [1]. In this study, we found that SMYD2 was related to the poor prognosis of patients with cervical cancer and regulated the proliferation of cervical cancer. We therefore provide a novel therapeutic target, SMYD2, which has a good prospect on cervical cancer treatment.

Post-translational modifications affect the function of proteins and are involved in various cell process [23]. Our study confirmed a methyltransferase, SMYD2, was involved the development of cervical cancer, and this means of regulation is most likely achieved by influencing transcription of downstream genes. Methylation of lysine on histones is thought one of the basal modifications for global gene transcription [24]. Abnormalities in the post-translational modifications of proteins could alter the physiological activity of cells, such as promoting or inhibiting tumor growth [25]. Series of methyltransferases involved in the post-translational modification of proteins could affect the occurrence and development of tumors [26, 27]. This effect is often quite complex, which is jointly regulated by multiple target genes and related signal pathways. In view of the influence of SMYD2 on cervical cancer, the downstream regulation mechanism needs further study.

Interestingly, the SMYD family, as a class a histone transferase, could regulate the transcription of downstream genes and participate in the development of multiple tumors. SMYD3 was involved in the regulation of cell proliferation of gastric cancer and was positively associated with poor prognosis in this cancer [28]. SMYD4 was also reported to be a tumor suppressor of breast cancer [29]. In this study, we found that SMYD2 is closely related to cervical cancer and further confirmed its regulatory effect on the proliferation of cervical cancer cells. Our findings, together with other previous studies, confirmed the involvement of the SMYD family in the regulation of tumorigenesis. As a histone methyltransferase, SMYD family members could affect such processes as tumor proliferation, migration, invasion and apoptosis through the transcriptional regulation of downstream genes and various signaling pathways [30]. Of course, different SMYD family proteins are specific to the regulation of downstream genes and regulate tumor growth in different ways.

The role of SMYD2 in tumors has been confirmed by multiple studies, and SMYD2 is currently considered to be a novel oncogene. The regulatory effect of SMYD2 on different tumors is different. In triple negative breast cancer, PTPN13, the transcriptional target gene of SMYD2, could be phosphorylated by SMYD2 and link SMYD2 to breast cancer associated signaling pathways, such as ERK, and mTOR signaling pathways, further affected breast cancer growth [31]. SMYD2 were highly expressed in pancreatic cancer and knockdown of SMYD2 blocked pancreatic cancer growth by affecting inflammation and stress responses [32]. SMYD2 methylates β-catenin and further promotes its nuclear translocation and activation of Wnt signaling, which is involved the development of liver cancer [33]. SMYD2 overexpression also contributed to malignant outcome in the development of gastric cancer through the regulation of cell proliferation [14]. We also found the similar result in cervical cancer that SMYD2 could further affect tumor development by affecting the proliferation of tumor cells. However, it is still unclear which target genes are regulated by SMYD2 to affect the proliferation of tumor cells, and further studies are therefore needed.

## Conclusion

Cervical cancer is the most common gynecological malignancy with low terminal cure rate, and SMYD2 is highly expressed in multiple tumors and affects their occurrence and development. We first used the bioinformatics website to screen the data of cervical cancer in TCGA and found the high expression of SMYD2 in cervical cancer, and survival analysis found that the poorer survival rate in the SMYD2 high expression group than that in the low expression group. Herein, our study demonstrated that the expression of SMYD2 in patients with cervical cancer was associated with FIGO stage, tumor size and correlated with poor prognosis. Data showed that inhibition of SMYD2 expression in cervical cancer cell line Caski and Siha could dramatically block the proliferation of cervical cancer cells. Additionally, SMYD2-shRNA lentivirus infected remarkably inhibited tumorigenesis of cervical cancer cells in mice compared with the scramble group. Taken together, this study provides strong evidence of the involvement of SMYD2 in cervical tumor growth and indicates that it could have high potential as a therapeutic target of cervical cancer.

## Data Availability

The dataset supporting the conclusions of this article is included within the article.

## References

[1] Joo JH, Kim YS, Nam JH (2018). Prognostic significance of lymph node ratio in node-positive cervical cancer patients. Medicine.

[2] Wang J, Bai Z, Wang Z, Yu C (2016). Comparison of secular trends in cervical cancer mortality in China and the United States: an age-period-cohort analysis. Int J Environ Res Public Health..

[3] Valenti G, Vitale SG, Tropea A, Biondi A, Lagana AS (2017). Tumor markers of uterine cervical cancer: a new scenario to guide surgical practice?. Updates Surg.

[4] Chen Y, Miller C, Mosher R, Zhao X, Deeds J, Morrissey M, Bryant B, Yang D, Meyer R, Cronin F, Gostout BS, Smith-McCune K, Schlegel R (2003). Identification of cervical cancer markers by cDNA and tissue microarrays. Cancer Res.

[5] Sun R, Koubaa I, Limkin EJ, Dumas I, Bentivegna E, Castanon E, Gouy S, Baratiny C, Monnot F, Maroun P, Ammari S, Zareski E, Balleyguier C, Deutsch E, Morice P, Haie-Meder C, Chargari C (2018). Locally advanced cervical cancer with bladder invasion: clinical outcomes and predictive factors for vesicovaginal fistulae. Oncotarget.

[6] Derks M, Groenman FA, van Lonkhuijzen L, Schut PC, Westerveld H, van der Velden J, Kenter GG (2017). Completing or abandoning radical hysterectomy in early-stage lymph node-positive cervical cancer: impact on disease-free survival and treatment-related toxicity. Int J Gynecol Cancer.

[7] Chen CY, Liu TZ, Tseng WC, Lu FJ, Hung RP, Chen CH, Chen CH (2008). (−)-Anonaine induces apoptosis through Bax- and caspase-dependent pathways in human cervical cancer (HeLa) cells. Food Chem Toxicol.

[8] Li Y, Carlson E, Villarreal R, Meraz L, Pagan JA (2017). Cost-effectiveness of a patient navigation program to improve cervical cancer screening. Am J Manag Care.

[9] Abu-Farha M, Lambert JP, Al-Madhoun AS, Elisma F, Skerjanc IS, Figeys D (2008). The tale of two domains: proteomics and genomics analysis of SMYD2, a new histone methyltransferase. Mol Cell Proteomics.

[10] Toghill BJ, Saratzis A, Freeman PJ, Sylvius N, Bown MJ (2018). SMYD2 promoter DNA methylation is associated with abdominal aortic aneurysm (AAA) and SMYD2 expression in vascular smooth muscle cells. Clin Epigenet..

[11] Diehl F, Brown MA, van Amerongen MJ, Novoyatleva T, Wietelmann A, Harriss J, Ferrazzi F, Bottger T, Harvey RP, Tucker PW, Engel FB (2010). Cardiac deletion of Smyd2 is dispensable for mouse heart development. PLoS ONE.

[12] Sesé B, Barrero MJ, Fabregat MC, Sander V, Belmonte JC (2013). SMYD2 is induced during cell differentiation and participates in early development. Int J Dev Biol..

[13] Cho HS, Hayami S, Toyokawa G, Maejima K, Yamane Y, Suzuki T, Dohmae N, Kogure M, Kang D, Neal DE, Ponder BA, Yamaue H, Nakamura Y, Hamamoto R (2012). RB1 methylation by SMYD2 enhances cell cycle progression through an increase of RB1 phosphorylation. Neoplasia.

[14] Komatsu S, Ichikawa D, Hirajima S, Nagata H, Nishimura Y, Kawaguchi T, Miyamae M, Okajima W, Ohashi T, Konishi H, Shiozaki A, Fujiwara H, Okamoto K, Tsuda H, Imoto I, Inazawa J, Otsuji E (2015). Overexpression of SMYD2 contributes to malignant outcome in gastric cancer. Br J Cancer.

[15] Piao L, Kang D, Suzuki T, Masuda A, Dohmae N, Nakamura Y, Hamamoto R (2014). The histone methyltransferase SMYD2 methylates PARP1 and promotes poly(ADP-ribosyl)ation activity in cancer cells. Neoplasia..

[16] Sajjad A, Novoyatleva T, Vergarajauregui S, Troidl C, Schermuly RT, Tucker HO, Engel FB (1843). Lysine methyltransferase Smyd2 suppresses p53-dependent cardiomyocyte apoptosis. Biochim Biophys Acta.

[17] Nakakido M, Deng Z, Suzuki T, Dohmae N, Nakamura Y, Hamamoto R (2015). Dysregulation of AKT pathway by SMYD2-mediated lysine methylation on PTEN. Neoplasia.

[18] Wang R, Deng X, Yoshioka Y, Vougiouklakis T, Park JH, Suzuki T, Dohmae N, Ueda K, Hamamoto R, Nakamura Y (2017). Effects of SMYD2-mediated EML4-ALK methylation on the signaling pathway and growth in non-small-cell lung cancer cells. Cancer Sci.

[19] Bagislar S, Sabo A, Kress TR, Doni M, Nicoli P, Campaner S, Amati B (2016). Smyd2 is a Myc-regulated gene critical for MLL-AF9 induced leukemogenesis. Oncotarget.

[20] Chacko S (2014). Effect of structured teaching programme on VIA test for early detection and diagnosis of cervical cancer. Nurs J India.

[21] Molina R, Filella X, Augé JM, Bosch E, Torne A, Pahisa J, Lejarcegui JA, Rovirosa A, Mellado B, Ordi J, Biete A (2005). CYFRA 211 in patients with cervical cancer: comparison with SCC and CEA. Anticancer Res..

[22] Zamani N, Gilani MM, Zamani F, Zamani MH (2015). Utility of pelvic MRI and tumor markers HE4 and CA125 to predict depth of myometrial invasion and cervical involvement in endometrial cancer. J Fam Reprod Health..

[23] Steinberg SF (2018). Post-translational modifications at the ATP-positioning G-loop that regulate protein kinase activity. Pharmacol Res.

[24] Zhang X, Wen H, Shi X (2012). Lysine methylation: beyond histones. Acta Biochim Biophys Sin (Shanghai).

[25] Garg B, Giri B, Majumder K, Dudeja V, Banerjee S, Saluja A (2017). Modulation of post-translational modifications in beta-catenin and LRP6 inhibits Wnt signaling pathway in pancreatic cancer. Cancer Lett.

[26] Jiang C, He C, Wu Z, Li F, Xiao J (2018). Histone methyltransferase SETD2 regulates osteosarcoma cell growth and chemosensitivity by suppressing Wnt/beta-catenin signaling. Biochem Biophys Res Commun.

[27] Barcena-Varela M, Caruso S, Llerena S, Alvarez-Sola G, Uriarte I, Latasa MU, Urtasun R, Rebouissou S, Alvarez L, Jimenez M, Santamaria E, Rodriguez-Ortigosa C, Mazza G, Rombouts K, Jose-Eneriz ES, Rabal O, Agirre X, Iraburu M, Santos-Laso A, Banales JM, Zucman-Rossi J, Prosper F, Oyarzabal J, Berasain C, Avila MA, Fernandez-Barrena MG (2019). Dual targeting of histone methyltransferase G9a and DNA-methyltransferase 1 for the treatment of experimental hepatocellular carcinoma. Hepatology..

[28] Wang L, Wang QT, Liu YP, Dong QQ, Hu HJ, Miao Z, Li S, Liu Y, Zhou H, Zhang TC, Ma WJ, Luo XG (2017). ATM signaling pathway is implicated in the SMYD3-mediated proliferation and migration of gastric cancer cells. J Gastric Cancer.

[29] Hu L, Zhu YT, Qi C, Zhu YJ (2009). Identification of Smyd4 as a potential tumor suppressor gene involved in breast cancer development. Cancer Res.

[30] Al-Shar’i NA, Alnabulsi SM (2016). Explaining the autoinhibition of the SMYD enzyme family: a theoretical study. J Mol Graph Model.

[31] Li LX, Zhou JX, Calvet JP, Godwin AK, Jensen RA, Li X (2018). Lysine methyltransferase SMYD2 promotes triple negative breast cancer progression. Cell Death Dis.

[32] Reynoird N, Mazur PK, Stellfeld T, Flores NM, Lofgren SM, Carlson SM, Brambilla E, Hainaut P, Kaznowska EB, Arrowsmith CH, Khatri P, Stresemann C, Gozani O, Sage J (2016). Coordination of stress signals by the lysine methyltransferase SMYD2 promotes pancreatic cancer. Genes Dev.

[33] Deng X, Hamamoto R, Vougiouklakis T, Wang R, Yoshioka Y, Suzuki T, Dohmae N, Matsuo Y, Park JH, Nakamura Y (2017). Critical roles of SMYD2-mediated beta-catenin methylation for nuclear translocation and activation of Wnt signaling. Oncotarget.

